# Preoperative Patient-reported Physical Functioning and Postoperative Wearable-measured Physical Activity in Cancer Patients: A Secondary Analysis of the SCRUM-MONSTAR LIFELOG Study

**DOI:** 10.31662/jmaj.2026-0300

**Published:** 2026-08-14

**Authors:** Shugo Yajima, Shin Kobayashi, Tadayoshi Hashimoto, Yoshiaki Nakamura, Riu Yamashita, Toshihiro Misumi, Yasutoshi Sakamoto, Takao Fujisawa, Mitsuho Imai, Taro Shibuki, Hideaki Bando, Hitoshi Masuda, Eiji Oki, Takayuki Yoshino

**Affiliations:** 1 Department of Urology, National Cancer Center Hospital East, Kashiwa, Japan; 2 Department of Hepatobiliary and Pancreatic Surgery, National Cancer Center Hospital East, Kashiwa, Japan; 3 Translational Research Support Office, National Cancer Center Hospital East, Kashiwa, Japan; 4 Department of Gastroenterology and Gastrointestinal Oncology, National Cancer Center Hospital East, Kashiwa, Japan; 5 Department of Oncology, University of Oxford, Oxford, United Kingdom; 6 Division of Translational Informatics, Exploratory Oncology Research and Clinical Trial Center, National Cancer Center, Kashiwa, Japan; 7 Department of Data Science, National Cancer Center Hospital East, Kashiwa, Japan; 8 Department of Head and Neck Medical Oncology, National Cancer Center Hospital East, Kashiwa, Japan; 9 Department of Advanced Medicine and Innovative Technology, Kyushu University Hospital, Fukuoka, Japan

**Keywords:** accelerometry, geriatric assessment, neoplasms, exercise, postoperative period

## Abstract

**Introduction:**

It remains unclear whether preoperative patient-reported physical functioning (PF) correlates with postoperative wearable-measured physical activity in cancer patients. Clarifying this relationship could indicate whether brief self-report items capture information relevant to subsequent postoperative outpatient activity.

**Methods:**

We analyzed 119 postoperative cancer patients from the SCRUM-MONSTAR LIFELOG study using four preoperative Medical Outcomes Study Short Form-36 (SF-36) PF items (stair climbing, vigorous activity, moderate activity, and carrying items; composite score 0-8), the Geriatric-8 (G8) screening tool, and wrist-based wearable accelerometry data. Spearman rank correlations, Wilcoxon rank-sum tests, and overlap-weighted analyses adjusted for clinical covariates were performed. Exploratory residual-based analyses examined the discordance between preoperative assessment and postoperative activity.

**Results:**

Patients with full PF (PF = 8; n = 53) versus impaired PF (PF < 8; n = 66) showed lower sedentary time (median 83.6% vs 87.3%; p = 0.031), higher moderate-to-vigorous physical activity (MVPA; 4.1% vs 2.7%; p = 0.015), and higher mean acceleration (21.8 vs 16.8 milligravity units; p = 0.044). All four SF-36 PF items showed consistent associations with sedentary time (Spearman ρ: −0.24 to −0.27) and MVPA (ρ: +0.23 to +0.29). G8-vulnerable patients showed higher sedentary time (87.2% vs 82.7%; p = 0.021) and lower MVPA (2.9% vs 4.1%; p = 0.045). After overlap-weighted adjustment, differences were attenuated. In exploratory analyses, age showed the most consistent, although weak, association with the discrepancy between preoperative assessment and postoperative activity.

**Conclusions:**

The preoperative SF-36 PF items showed weak but consistent unadjusted associations with postoperative physical activity, although attenuated after adjustment for baseline clinical covariates. These findings suggest that brief preoperative self-report items may capture information relevant to subsequent physical activity behavior in cancer patients, with group-level differences partly reflecting shared clinical context.

## Introduction

Functional status is a central determinant of treatment tolerance and postoperative recovery in patients with cancer. Patient-reported outcome instruments, particularly the Physical Functioning (PF) subscale of the Medical Outcomes Study Short Form-36 (SF-36), have been widely used to capture self-perceived physical capacity in oncological settings ^(1)^. The SF-36 PF subscale assesses the degree to which health limits activities ranging from vigorous exertion to basic daily tasks, and has demonstrated validity and responsiveness across diverse patient populations ^(1), (2)^.

Wearable accelerometry provides an objective, continuous measure of physical activity (PA) in free-living conditions, complementing subjective questionnaire-based assessments ^(3)^. Common accelerometer-derived metrics include sedentary time, moderate-to-vigorous PA (MVPA) percentage, and mean acceleration. The adoption of wearable PA monitors has expanded among middle-aged and older adults, in whom their use has been associated with higher PA levels and lower locomotive syndrome risk ^(4)^.

Despite the complementary use of patient-reported and objective activity measures in cancer care, whether preoperative self-reported PF correlates with postoperative wearable-measured activity has not been well characterized. Such a cross-method, cross-time association would have practical implications: brief preoperative questionnaire items could serve as accessible correlates of subsequent postoperative outpatient PA, potentially helping to identify patients likely to have reduced postoperative activity. We therefore conducted a secondary analysis of the SCRUM-MONSTAR LIFELOG study ^(5)^, a prospective registry collecting wearable device data in postoperative cancer patients, to examine whether the four preoperative SF-36 PF items correlate with postoperative wearable-measured PA. For comparison, we also examined associations with the Geriatric-8 (G8) screening tool ^(6), (7), (8)^, a validated geriatric assessment instrument whose prognostic value in older patients with cancer has been systematically reviewed, and which has been recommended as a frailty screening tool for older surgical patients ^(9)^.

## Materials and Methods

### Study design and participants

The SCRUM-MONSTAR LIFELOG study ^(5)^ is an ancillary study of the SCRUM-Japan MONSTAR-SCREEN-3 registry, a nationwide multicenter prospective registry that enrolls patients with resectable solid tumors after obtaining written informed consent preoperatively ^(10)^. MONSTAR-SCREEN-3 participants who underwent curative-intent surgery at a single participating institution and provided additional written informed consent for the LIFELOG ancillary study between November 2024 and November 2025 were enrolled, provided they had both preoperative assessment data and postoperative wearable device data. The parent MONSTAR-SCREEN-3 registry and the LIFELOG ancillary study were approved by the Institutional Review Board of National Cancer Center Hospital East (No. 2023-354 and No. 2024-111, respectively). This study followed the Strengthening the Reporting of Observational Studies in Epidemiology (STROBE) guidelines for observational studies.

### Wearable device and PA metrics

Device wearing was initiated at 1 month after surgery, once participants had been discharged from hospital, were no longer bedridden, and were able to attend outpatient clinic visits. Starting from this time point, participants wore a wrist-based wearable device (mSafety™, Sony Network Communications Inc., Tokyo, Japan) ^(11)^ for a target duration of 1 month. Participants were instructed to wear the device during waking hours only and to remove it before sleep. Three-axis accelerometer data sampled at 32 Hz were processed using a custom R pipeline, implementing algorithms compliant with the Generation of GeneRalized PA Indicators from Raw accelerometer data framework, adapted for the mSafety device ^(12)^. Processing included autocalibration to local gravity based on static windows, calculation of the Euclidean Norm Minus One (ENMO) expressed in milligravity units (mg), aggregation of raw signals into 5-second epochs aligned to clock time, and detection of non-wear time using a 60-minute moving window based on axis-specific thresholds (standard deviation <13 mg or value range <50 mg on at least two axes) ^(12)^. Epochs flagged as non-wear, including overnight device removal, were excluded; all PA metrics were therefore derived from valid wear time corresponding to daytime wear. Each valid wear-time epoch was classified by its mean ENMO as sedentary (<40 mg), light (40 to <100 mg), moderate (100 to <400 mg), or vigorous (≥400 mg) using established wrist-worn cut-offs ^(13), (14)^. Participant-level metrics were obtained by averaging across valid wear days: (1) sedentary time percentage; (2) MVPA percentage (epochs ≥100 mg, combining moderate and vigorous categories); (3) mean acceleration—the average ENMO (mg) across valid wear time; and (4) maximum MVPA bout duration—the longest continuous run of consecutive MVPA-classified epochs, expressed in minutes. A wear-time adequacy criterion (≥3 valid days with ≥10 hours of daily wear) was used as a data-quality indicator, not an exclusion criterion.

### SF-36 PF items

Preoperative PF was assessed via patient self-report collected by trained nurses using four items derived from the SF-36 PF subscale ^(1), (2)^, with item wording and response options adapted into Japanese for routine preoperative clinical use at our institution: (1) climbing one or more flights of stairs, (2) vigorous activities (e.g., running, lifting heavy objects, or participating in strenuous sports), (3) moderate activities (e.g., cleaning the house or garden or walking for 1-2 hours), and (4) lifting or carrying moderately heavy items (e.g., a 5-kg bag of rice). Each item was rated on a 3-level ordinal scale rescaled to 0-2 for this analysis (0 = limited a lot, 1 = limited a little, and 2 = not limited at all) rather than the standard SF-36 1-3 scoring. An SF-36-derived PF composite score (range 0-8) was derived by summing the four items; patients scoring 8 were categorized as having a full PF score (PF = 8), and those scoring below 8 as having an impaired PF score (PF < 8). The resulting PF group contrast (full vs impaired) was used in the group-level analyses. These four items are those routinely administered as part of the preoperative geriatric assessment at our institution and were selected to span a gradient of physical demand, from vigorous activity to basic mobility; they do not constitute a formally validated abbreviation of the SF-36 PF subscale. Because the composite showed a pronounced ceiling effect, it was dichotomized into a full score (PF = 8) versus any reported limitation (PF < 8) for a clinically interpretable contrast; this threshold is pragmatic rather than a validated cut-off. To avoid relying solely on dichotomization, the composite and individual items were also analyzed as continuous or ordinal variables using rank correlations, and an exploratory analysis treated the composite as a continuous predictor (see Statistical analysis).

### G8 assessment

The G8 was assessed preoperatively by trained nurses, concurrently with the SF-36 PF items. The G8 comprises 8 items: food intake decline, weight loss, mobility, neuropsychological status, body mass index (BMI), polypharmacy, self-perceived health, and age category (total score 0-17). Patients were categorized as G8-fit (G8 > 14) or G8-vulnerable (G8 ≤ 14) according to the validated threshold ^(6), (7), (8)^.

### Statistical analysis

Baseline characteristics were compared using Wilcoxon rank-sum tests for continuous variables and chi-square or Fisher’s exact tests for categorical variables. PA metrics are presented as median (interquartile range [IQR]) and compared between groups using Wilcoxon rank-sum tests. Spearman rank and Kendall rank correlations quantified associations between PA metrics and continuous scores. Bootstrap confidence intervals (CIs; B = 1,000) and permutation p values (n = 1,000) were used for overlap-weighted (OW) inference. To assess whether group differences were attributable to differences in baseline clinical characteristics, OW analyses were performed using propensity score-based weighting for the average treatment effect in the overlap population ^(15)^. For G8 analyses, covariates included cancer type, sex, and disease stage; age was excluded because it constitutes a component of the G8 score. For PF group analyses, covariates included age, cancer type, sex, and disease stage. Recurrence-free survival, defined as the time from surgery to confirmed disease recurrence with censoring at last follow-up, was examined as an exploratory end point using Kaplan–Meier estimation, log-rank tests, and univariable Cox proportional hazards regression with hazard ratios (HRs). As a further exploratory analysis, each of the three volume-related PA metrics (sedentary time, MVPA, and mean acceleration) was regressed on the SF-36-derived PF composite score, and the residuals (observed − predicted) were examined; maximum MVPA bout duration was excluded as it was unrelated to the preoperative measures. Associations between residuals and continuous factors (age and G8 score) were assessed with Spearman correlations and with binary factors by Wilcoxon rank-sum tests. Using mean-acceleration residuals, patients were grouped into under-performer, concordant, and over-performer tertiles; under- and over-performers were then compared using Wilcoxon rank-sum tests for continuous variables (with disease stage treated as an ordinal score) and Fisher’s exact tests for categorical variables (sex, cancer type, chemotherapy, and recurrence). Age–activity associations were additionally examined within the full–PF-score subgroup. No adjustment was made for multiple comparisons, and these analyses were interpreted descriptively. All analyses were conducted using R version 4.5.3 (R Foundation for Statistical Computing, Vienna, Austria). Two-sided p < 0.05 was considered statistically significant.

## Results

### Participants

Between November 2024 and November 2025, 127 patients were enrolled in the SCRUM-MONSTAR LIFELOG study. Wearable device data were available in 123 patients, and both preoperative G8 and SF-36 PF assessments were available in 123 patients. Because the four patients without preoperative assessments and the four patients without device data were non-overlapping, the final analysis dataset comprised 119 patients. Of these 119 patients, 110 (92.4%) met the wear-time adequacy criterion (≥3 valid days with ≥10 hours of daily wear); all 119 were included in the primary analyses. Participant characteristics are presented in Table 1. The most common tumor types were colorectal (23.5%), urothelial (20.2%), hepatocellular (10.9%), renal cell (9.2%), and gastric (8.4%) cancer. Forty-three patients (36.1%) were classified as G8-fit (G8 > 14) and 76 (63.9%) as G8-vulnerable (G8 ≤ 14). The G8-vulnerable group was older (median 73.0 years [IQR 63.2-79.8 years] vs 69.5 years [IQR 59.5-74.9 years]; p = 0.018) and had more advanced disease stage (Fisher’s exact, p = 0.019). Fifty-three patients (44.5%) had a full PF score (PF = 8) and 66 (55.5%) had an impaired PF score (PF < 8). G8-fit patients had higher rates of full PF score than G8-vulnerable patients (60.5% vs 35.5%; p = 0.009). Patients with a full PF score were younger than those with an impaired PF score (median 65.5 years [IQR 55.3-72.0 years] vs 75.8 years [IQR 66.9-80.2 years]; p < 0.001) and had higher G8 scores (14.0 vs 13.0; p < 0.001); the distribution of cancer types also differed between PF groups (p = 0.016), whereas sex and disease stage were comparable (Table 1).

**Table 1.** Participant Characteristics and Postoperative PA Metrics (N = 119).

Characteristic	Overall (N = 119)	G8-fit (n = 43)	G8-vulnerable (n = 76)	p Value	Full PF (n = 53)	Impaired PF (n = 66)	p Value
Age, years	70.2 (61.9-78.0)	69.5 (59.5-74.9)	73.0 (63.2-79.8)	0.018	65.5 (55.3-72.0)	75.8 (66.9-80.2)	<0.001
Male sex, n (%)	69 (58.0)	25 (58.1)	44 (57.9)	0.979	30 (56.6)	39 (59.1)	0.785
Cancer type, n (%)							
Colorectal	28 (23.5)	10 (23.3)	18 (23.7)	0.49	15 (28.3)	13 (19.7)	0.016
Urothelial	24 (20.2)	5 (11.6)	19 (25.0)		9 (17.0)	15 (22.7)	
Hepatocellular	13 (10.9)	7 (16.3)	6 (7.9)		2 (3.8)	11 (16.7)	
Renal cell	11 (9.2)	4 (9.3)	7 (9.2)		2 (3.8)	9 (13.6)	
Gastric	10 (8.4)	4 (9.3)	6 (7.9)		4 (7.5)	6 (9.1)	
Other	33 (27.7)	13 (30.2)	20 (26.3)		21 (39.6)	12 (18.2)	
Disease stage, n (%)							
0	7 (5.9)	1 (2.3)	6 (7.9)	0.019	3 (5.7)	4 (6.1)	0.625
I	21 (17.6)	10 (23.3)	11 (14.5)		7 (13.2)	14 (21.2)	
II	35 (29.4)	19 (44.2)	16 (21.1)		19 (35.8)	16 (24.2)	
III	48 (40.3)	11 (25.6)	37 (48.7)		20 (37.7)	28 (42.4)	
IV	8 (6.7)	2 (4.7)	6 (7.9)		4 (7.5)	4 (6.1)	
G8 score (0-17)	14.0 (12.0-15.0)	15.0 (15.0-16.0)	12.5 (11.5-13.6)	<0.001	14.0 (13.0-15.5)	13.0 (11.5-14.4)	<0.001
SF-36-derived PF composite score (0-8)	7.0 (6.0-8.0)	8.0 (7.0-8.0)	7.0 (6.0-8.0)	0.009	—	—	—
Full PF score (PF = 8), n (%)	53 (44.5)	26 (60.5)	27 (35.5)	0.009	—	—	—
SF-36 PF items: score = 2 (not limited at all), n (%)							
Stair climbing	106 (89.1)	39 (90.7)	67 (88.2)	0.637	—	—	—
Vigorous activity	54 (45.4)	26 (60.5)	28 (36.8)	0.023	—	—	—
Moderate activity	100 (84.0)	39 (90.7)	61 (80.3)	0.284	—	—	—
Carrying items	101 (84.9)	39 (90.7)	62 (81.6)	0.504	—	—	—
Postoperative PA metrics							
Sedentary time (%)	85.6 (79.2-91.1)	82.7 (78.3-87.5)	87.2 (80.9-92.1)	0.021	83.6 (77.9-89.5)	87.3 (80.4-92.7)	0.031
MVPA (%)	3.4 (1.8-6.4)	4.1 (2.4-6.7)	2.9 (1.6-5.7)	0.045	4.1 (2.7-6.8)	2.7 (1.4-5.2)	0.015
Mean acceleration (ENMO, mg)	19.1 (12.9-25.5)	21.8 (16.9-25.6)	16.8 (11.6-25.4)	0.071	21.8 (15.7-26.0)	16.8 (10.7-24.9)	0.044
Max MVPA bout duration (min)	6.3 (2.7-13.5)	6.3 (2.7-12.7)	6.4 (2.6-14.5)	0.836	6.0 (3.2-13.4)	7.5 (1.9-13.6)	0.904

Continuous variables: median (IQR). Categorical variables: n (%). p values: Wilcoxon rank-sum (continuous); chi-square or Fisher’s exact (categorical). For the PF-group comparison, p values for the SF-36-derived PF composite score, the full PF score, and the four SF-36 PF items are not reported (—) because these variables define the PF groups. Cancer type was compared with the chi-square test and disease stage with Fisher’s exact test. ENMO: Euclidean Norm Minus One; G8: Geriatric-8 screening tool; IQR: interquartile range; mg: milligravity units; MVPA: moderate-to-vigorous physical activity; PA: physical activity; PF: physical functioning; SF-36 PF: four-item subset from the Medical Outcomes Study Short Form-36 health survey Physical Functioning subscale (the SF-36-derived PF composite score ranges 0-8).

### G8 status and postoperative PA

The G8 total score correlated with sedentary time (ρ = −0.31; p < 0.001), MVPA (ρ = +0.26; p = 0.005), and mean acceleration (ρ = +0.26; p = 0.004), whereas no correlation was observed with maximum MVPA bout duration (ρ = +0.01; p = 0.930; Figure 1). G8-vulnerable patients spent a greater proportion of wear time in sedentary behavior (87.2% [IQR 80.9-92.1] vs 82.7% [IQR 78.3-87.5]; p = 0.021) and had lower MVPA (2.9% [IQR 1.6-5.7] vs 4.1% [IQR 2.4-6.7]; p = 0.045), compared with G8-fit patients (Figure 2A, Table 2). Between-group differences in mean acceleration and maximum MVPA bout duration did not reach statistical significance (p = 0.071 and p = 0.836, respectively). Among G8 domain items, the age category and BMI items showed the most consistent associations with wearable-measured PA metrics (Supplementary Figure S1).

**Figure 1.**
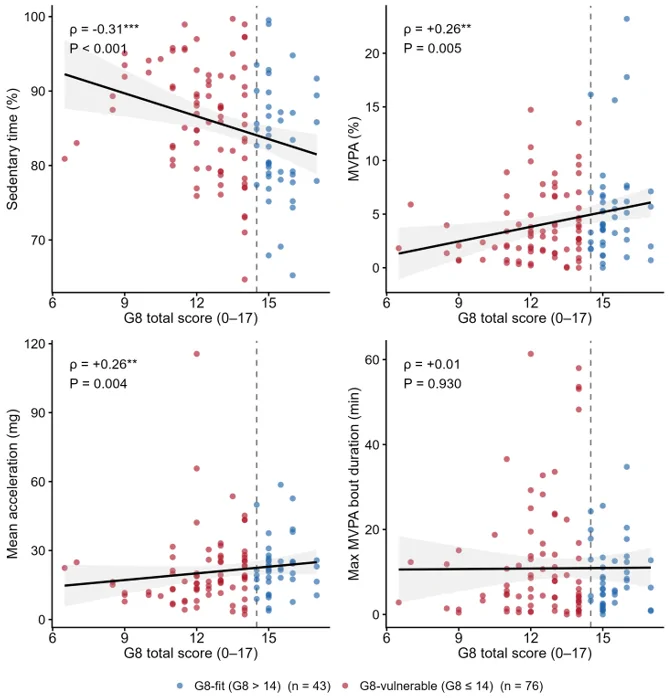
G8 total score and postoperative PA. Scatter plots showing the relationship between preoperative G8 total score (x-axis, 0-17) and each postoperative PA metric (y-axis). Blue: G8-fit (G8 > 14; n = 43); red: G8-vulnerable (G8 ≤ 14; n = 76). Vertical dashed line: classification threshold. Solid line: fitted linear reference line (95% CI shaded) shown for visualization only; statistics reported are Spearman rank correlations. Spearman ρ and p value per panel. ρ: Spearman rank correlation coefficient; CI: confidence interval; G8: Geriatric-8 screening tool; mg: milligravity units; MVPA: moderate-to-vigorous physical activity; PA: physical activity.

**Figure 2.**
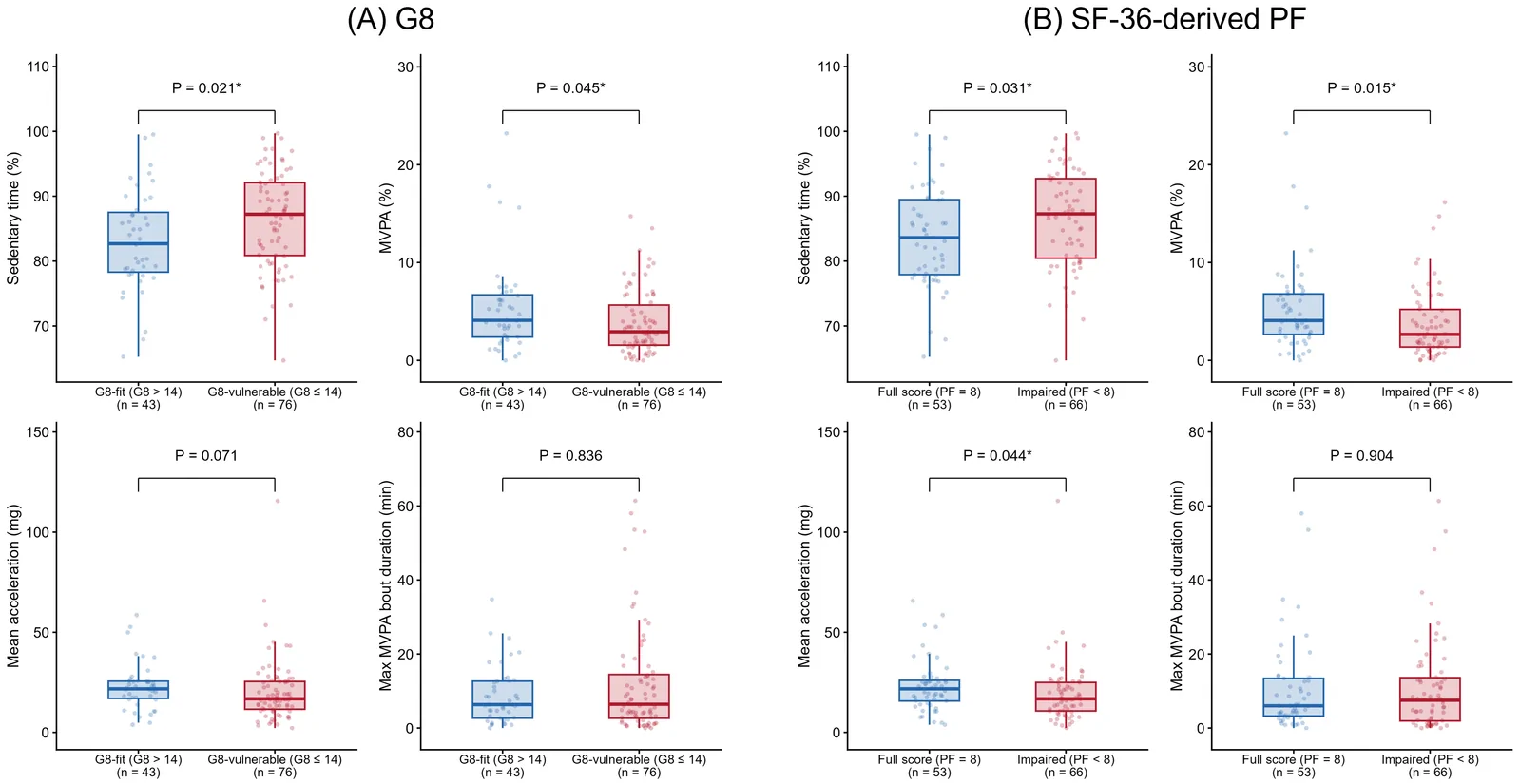
Postoperative PA by preoperative assessment group. Box plots comparing postoperative PA metrics between groups. Boxes: interquartile range; horizontal lines: medians; individual data points shown with jitter. p values: Wilcoxon rank-sum test. (A) G8 category: G8-fit (blue; n = 43) versus G8-vulnerable (red; n = 76). (B) PF group: full PF score (PF = 8; blue; n = 53) versus impaired PF score (PF < 8; red; n = 66). G8: Geriatric-8 screening tool; mg: milligravity units; MVPA: moderate-to-vigorous physical activity; PA: physical activity; PF: physical functioning; SF-36: Medical Outcomes Study Short Form-36 health survey.

**Table 2.** Postoperative PA by Preoperative Assessment Group.

PA metric	G8-fit (n = 43)	G8-vulnerable (n = 76)	p Value	OW diff [95% CI]
(adj: cancer type, sex, disease stage)				
Sedentary time (%)	82.7 (78.3-87.5)	87.2 (80.9-92.1)	0.021*	−2.77 pp [−6.17 to +0.87]; p = 0.068
MVPA (%)	4.1 (2.4-6.7)	2.9 (1.6-5.7)	0.045*	+1.27 pp [−0.45 to +3.12]; p = 0.123
Mean acceleration (ENMO, mg)	21.8 (16.9-25.6)	16.8 (11.6-25.4)	0.071	+1.87 mg [−3.47 to +7.71]; p = 0.518
Max MVPA bout duration (min)	6.3 (2.7-12.7)	6.4 (2.6-14.5)	0.836	−3.21 min [−7.64 to +1.00]; p = 0.213
PA metric	Full PF score (n = 53)	Impaired PF score (n = 66)	p Value	OW diff [95% CI]
(adj: age, cancer type, sex, disease stage)				
Sedentary time (%)	83.6 (77.9-89.5)	87.3 (80.4-92.7)	0.031*	−0.79 pp [−4.52 to +2.86]; p = 0.574
MVPA (%)	4.1 (2.7-6.8)	2.7 (1.4-5.2)	0.015*	+0.16 pp [−1.87 to +2.08]; p = 0.826
Mean acceleration (ENMO, mg)	21.8 (15.7-26.0)	16.8 (10.7-24.9)	0.044*	−1.62 mg [−10.51 to +5.52]; p = 0.565
Max MVPA bout duration (min)	6.0 (3.2-13.4)	7.5 (1.9-13.6)	0.904	−1.57 min [−8.35 to +6.36]; p = 0.520

Values: median (IQR). p values: Wilcoxon rank-sum. *p < 0.05. OW diff: overlap-weighted mean difference (high-function group − low-function group), expressed in percentage points (pp) for sedentary time and MVPA, in mg for mean acceleration, and in minutes for maximum MVPA bout duration; 95% CI: bootstrap percentile (B = 1,000); p: permutation p value (n = 1,000). adj: adjusted covariates; CI: confidence interval; ENMO: Euclidean Norm Minus One; IQR: interquartile range; mg: milligravity units; MVPA: moderate-to-vigorous physical activity; OW: overlap weighting; PA: physical activity; PF: physical functioning; pp: percentage points.

### SF-36 PF items and postoperative PA

Patients with a full PF score (PF = 8; n = 53) showed lower sedentary time (median 83.6% [IQR 77.9-89.5] vs 87.3% [IQR 80.4-92.7]; p = 0.031), higher MVPA (4.1% [IQR 2.7-6.8] vs 2.7% [IQR 1.4-5.2]; p = 0.015), and higher mean acceleration (21.8 mg [IQR 15.7-26.0] vs 16.8 mg [IQR 10.7-24.9]; p = 0.044) than those with an impaired PF score (Figure 2B, Table 2). Maximum MVPA bout duration did not differ significantly (p = 0.904). The SF-36-derived PF composite score was consistently associated with sedentary time (ρ = −0.28; p = 0.002) and MVPA (ρ = +0.30; p = 0.001). All four SF-36 PF items showed consistent associations with sedentary time (ρ range: −0.24 to −0.27; all p < 0.01) and MVPA (ρ range: +0.23 to +0.29; all p < 0.05), corroborated by Kendall rank correlations (Figure 3).

**Figure 3.**
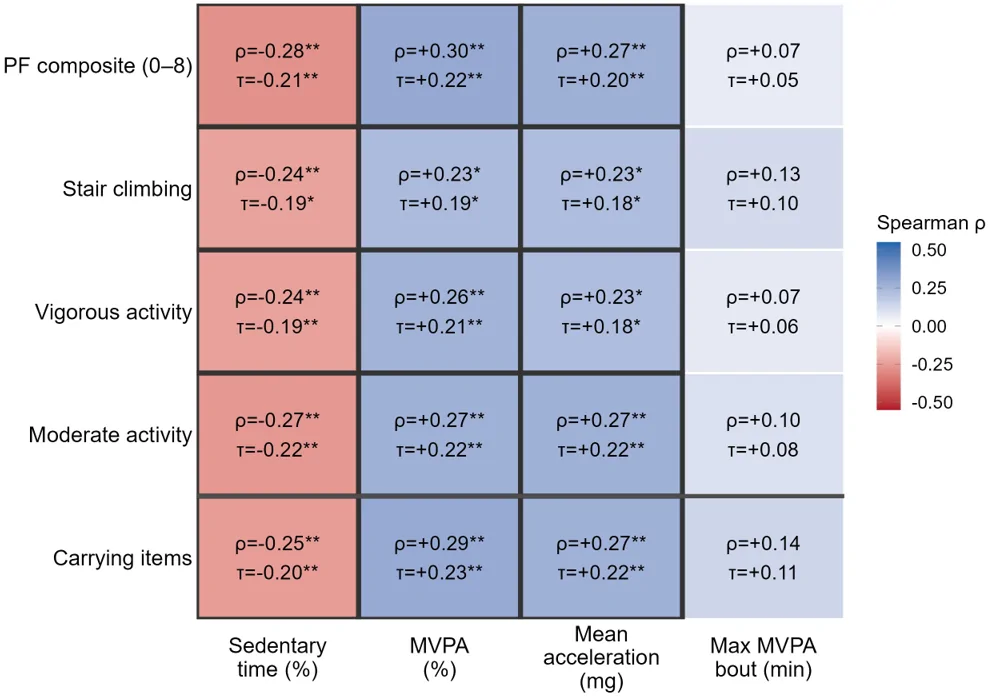
Spearman and Kendall correlations: SF-36 PF items and PA metrics. Each cell shows Spearman ρ (upper) and Kendall τ (lower). Top row: SF-36-derived PF composite score (0-8), separated from the four individual items (below) by a thicker horizontal border. Columns: four PA metrics. Cell color: direction and magnitude of Spearman ρ. Bold borders around cells: p < 0.05 (Spearman). *p < 0.05; **p < 0.01; ***p < 0.001. N = 119. ρ: Spearman rank correlation coefficient; τ: Kendall rank correlation coefficient; mg: milligravity units; MVPA: moderate-to-vigorous physical activity; PA: physical activity; PF: physical functioning; SF-36: Medical Outcomes Study Short Form-36 health survey.

### OW analyses

After overlap weighting (cancer type, sex, and disease stage), G8 differences in sedentary time (OW difference: −2.77 percentage points (pp) [95% CI −6.17 to +0.87]; permutation p = 0.068) and MVPA (+1.27 pp [−0.45 to +3.12]; p = 0.123) were attenuated and were no longer statistically significant. With additional adjustment for age in the PF analyses, OW differences in sedentary time (−0.79 pp [−4.52 to +2.86]; p = 0.574) and MVPA (+0.16 pp [−1.87 to +2.08]; p = 0.826) were also attenuated. For mean acceleration in the SF-36 PF comparison, the OW difference in arithmetic means was opposite in direction to the unadjusted median difference as shown in Table 2. The 95% CI was wide and included zero, indicating substantial uncertainty after weighting.

### Exploratory analysis: recurrence-free survival

Exploratory analysis was conducted in the 119-patient dataset (13 recurrence events; median follow-up 3.4 months [range 0.9-19.2 months]). Kaplan–Meier curves by G8 category and PF group are presented in Supplementary Figure S2 and S3, with univariable Cox proportional hazards regression results in Supplementary Table S1. In univariable Cox regression, G8 category was not associated with recurrence-free survival (HR for G8-vulnerable vs G8-fit: 1.73 [95% CI 0.47-6.33]; Wald p = 0.405). Among PF metrics, the SF-36-derived PF composite score was associated with recurrence risk in the direction of lower scores corresponding to higher risk (HR per 1 point: 0.73 [95% CI 0.56-0.96]; Wald p = 0.023), and the PF group comparison was directionally consistent (impaired vs full PF score: HR 4.16 [95% CI 0.92-18.81]; Wald p = 0.064). Given the limited event count, these findings should be interpreted as hypothesis-generating only.

### Exploratory analysis: discordance between preoperative functional assessment and postoperative activity

To identify patients whose postoperative activity diverged from that expected from their preoperative function, we examined residuals from regression of each of the three volume-related PA metrics on the SF-36-derived PF composite score. Among patients with similar preoperative function (median PF composite 7.0 in each residual tertile), observed activity varied widely (median mean acceleration 10.6, 19.2, and 27.9 mg across tertiles). Age showed the most consistent association with the residual (sedentary time ρ = +0.20, p = 0.026; MVPA ρ = −0.18, p = 0.045; and mean acceleration ρ = −0.20, p = 0.031; Supplementary Figure S4), older patients being less active than predicted. A directionally similar but non-significant pattern was seen within the full–PF-score (ceiling) subgroup (mean acceleration ρ = −0.27, p = 0.054; sedentary time ρ = +0.22, p = 0.118; Supplementary Figure S5). Under-performers (lowest mean-acceleration residual tertile) were numerically older (median 74.7 vs 69.1 years) and had lower G8 scores (12.5 vs 14.0) than over-performers, although these between-group comparisons were imprecise; no clear differences were observed in sex, cancer type, disease stage, chemotherapy, or recurrence (Supplementary Table S2); procedure-specific data and postoperative complications were unavailable. These associations were weak, were not adjusted for multiple comparisons, and should be regarded as hypothesis-generating.

## Discussion

In this secondary analysis of the SCRUM-MONSTAR LIFELOG study, the four preoperative SF-36 PF items showed weak but consistent unadjusted associations with wearable-measured PA during the postoperative outpatient period. Patients who reported better preoperative PF showed lower sedentary time, higher MVPA, and higher mean acceleration after surgery. These associations were directionally concordant with those for the G8 screening tool.

A notable feature is the cross-method, cross-time nature of the association: a questionnaire administered before surgery was associated with wearable-measured activity during the early outpatient period afterward, suggesting that self-reported functional status captures some information about physical capacity, although the associations were weak in magnitude.

The SF-36 PF subscale has been validated across a range of chronic disease populations ^(1), (2)^. The four items span a gradient of physical demand, and their consistent associations with sedentary time and MVPA, corroborated by both Spearman and Kendall rank correlations, support coherence across the items. The correlation magnitudes (Spearman ρ approximately 0.24-0.31) are weak, consistent with the broader literature on self-reported versus wearable-measured activity, where related but distinct constructs correlate imperfectly.

After OW adjustment, the group-level differences were attenuated and no longer statistically significant, indicating that part of the unadjusted association is attributable to shared clinical characteristics, particularly age and disease stage, and underscoring the importance of accounting for clinical context.

In the exploratory discordance analysis, age showed the most consistent association with the divergence between predicted and observed activity, whereas sex, tumor type, disease stage, chemotherapy, and recurrence did not. A directionally similar, though imprecise, age-related pattern was seen even among patients reporting maximal PF, suggesting that self-reported PF may overestimate subsequent activity in some older patients, in keeping with the ceiling effect and the attenuation observed after overlap weighting. The opposite direction of the OW mean difference in mean acceleration relative to the unadjusted median difference may partly reflect both covariate weighting and the use of arithmetic means rather than medians in a skewed distribution; however, the weighted estimate was imprecise. Clinically, preoperative PF items alone are unlikely to identify older patients at risk of low postoperative activity and may be most informative when combined with age or a broader geriatric screening tool such as the G8; if confirmed, brief assessment might help flag patients, particularly older patients, for prehabilitation, postoperative rehabilitation, or closer activity monitoring. However, clinically meaningful thresholds for these wearable-derived metrics have not been established in heterogeneous postoperative cancer populations, the effect sizes were modest, and the clinical importance of the observed unadjusted differences therefore remains uncertain and would require prospective evaluation.

Several limitations should be noted. First, PA was measured only postoperatively, beginning approximately 1 month after surgery; no preoperative or immediate-postoperative data were available; so, we cannot determine whether the associations reflect preoperative function or pre-existing habitual activity, nor can we capture the early recovery trajectory or complications. This limits causal interpretation, and future studies should ideally initiate wearable monitoring preoperatively and continue through the immediate postoperative period. Second, the four items were adapted into Japanese for routine clinical use and differ from the validated SF-36 v2 Japanese version; the derived composite is not the standardized SF-36 PF score and has not been formally validated, so comparisons with published SF-36 PF scores should be cautious. Third, a ceiling effect was evident, particularly for stair climbing (89.1% scored at the maximum), and the PF = 8 versus PF < 8 dichotomization, adopted for interpretability and not a validated cut-off, may reduce statistical power; reassuringly, the continuous and residual-based analyses were directionally concordant. Fourth, the cohort comprised diverse tumor types and, by extension, surgical procedures of differing invasiveness; although cancer type was adjusted for, procedure-specific variables and postoperative complications were not available, so residual confounding by surgical invasiveness cannot be excluded. Finally, follow-up was short (median 3.4 months, 13 events), precluding multivariable survival analysis, the cohort was from a single institution, and whether these associations extend to longer-term activity warrants further investigation.

In conclusion, the four preoperative SF-36 PF items showed consistent but weak unadjusted associations with wearable-measured postoperative PA, attenuated after adjustment for baseline clinical covariates. These findings support the concept that preoperative patient-reported functional assessment may capture information about subsequent PA behavior in cancer patients, while recognizing that this information partly overlaps with routinely collected clinical context such as age and disease stage.

## Article Information

### Author Contributions

Conceptualization: Shugo Yajima, Shin Kobayashi, Tadayoshi Hashimoto, and Takayuki Yoshino; Data curation: Shugo Yajima, Shin Kobayashi, Riu Yamashita, and Toshihiro Misumi; Formal analysis: Shin Kobayashi, Riu Yamashita, and Toshihiro Misumi; Funding acquisition: Shugo Yajima and Shin Kobayashi; Investigation: Shugo Yajima, Shin Kobayashi, Riu Yamashita, Toshihiro Misumi, Yasutoshi Sakamoto, Takao Fujisawa, Mitsuho Imai, and Taro Shibuki; Methodology: Shugo Yajima, Shin Kobayashi, Tadayoshi Hashimoto, and Takayuki Yoshino; Project administration: Shugo Yajima and Shin Kobayashi; Resources: Yasutoshi Sakamoto, Takao Fujisawa, Mitsuho Imai, and Taro Shibuki; Supervision: Shin Kobayashi, Yoshiaki Nakamura, Hideaki Bando, Hitoshi Masuda, Eiji Oki, and Takayuki Yoshino; Validation: Riu Yamashita and Toshihiro Misumi; Visualization: Shugo Yajima; Writing—original draft: Shugo Yajima and Shin Kobayashi; and Writing—review & editing: all authors.

Shugo Yajima and Shin Kobayashi had full access to all data in the study and take responsibility for the integrity of the data and the accuracy of the data analysis.

### Conflicts of Interest

Dr. Tadayoshi Hashimoto reports honoraria from CytoGen, Myriad Genetics, Guardant Health, Miyarisan, and Takata Pharmaceutical, and an advisory fee from Guardant Health, all outside the submitted work. Dr. Yoshiaki Nakamura reports personal fees from Guardant Health Pte, Ltd., Natera, Inc., Roche, Ltd., Premo Partners, Inc., Takeda Pharmaceutical, Exact Sciences Corporation, Gilead Sciences, Inc., MSD, Eisai Co., Ltd., Zeria Pharmaceutical Co., Ltd., Miyarisan Pharmaceutical Co., Ltd., Merck Biopharma Co., Ltd., CareNet, Inc., Hisamitsu Pharmaceutical Co., Inc., Taiho Pharmaceutical Co., Ltd., Becton, Dickinson and Company, and Guardant Health Japan Corp.; grants and personal fees from Seagen, Inc., Daiichi Sankyo Co., Ltd., and Chugai Pharmaceutical Co., Ltd.; and grants from Genomedia Inc., Guardant Health AMEA, Inc., Guardant Health, Inc., Tempus Labs, Inc., and Roche Diagnostics K.K., all outside the submitted work. Dr. Takao Fujisawa reports personal fees from Amelieff and AstraZeneca, outside the submitted work. Dr. Mitsuho Imai reports personal fees from Caris Life Sciences, Guardant Health, Inc., and Sumitomo Corp, outside the submitted work. Dr. Toshihiro Misumi reports honoraria from Chugai and AstraZeneca, outside the submitted work. Dr. Hideaki Bando reports personal fees from Ono Pharmaceutical, Taiho Pharmaceutical, and Eli Lilly Japan K.K., outside the submitted work. Dr. Eiji Oki reports research funding from Guardant Health and honoraria from Ono, Takeda, Bayer, Chugai, Taiho, Eli Lilly, and Bristol Myers Squibb, outside the submitted work. Dr. Takayuki Yoshino reports personal fees from Chugai Pharmaceutical Co., Ltd., Takeda Pharmaceutical, Merck Biopharma Co., Ltd., Bayer Yakuhin, Ono Pharmaceutical, MSD, and Sumitomo Corp and grants from Amgen, Chugai Pharmaceutical, Daiichi Sankyo, Eisai Co., Ltd., FALCO Biosystems, Genomedia, Molecular Health, MSD, Nippon Boehringer Ingelheim Co., Ltd., Ono Pharmaceutical, Pfizer, Roche Diagnostics, Sanofi, Sysmex, and Taiho Pharmaceutical, all outside the submitted work. Drs. Shugo Yajima, Shin Kobayashi, Riu Yamashita, Yasutoshi Sakamoto, Taro Shibuki, and Hitoshi Masuda declare that there are no competing interests.

### Ethics Statement

The parent SCRUM-Japan MONSTAR-SCREEN-3 registry (No. 2023-354) and the SCRUM-MONSTAR LIFELOG ancillary study (No. 2024-111) were approved by the Institutional Review Board of National Cancer Center Hospital East. All participants provided written informed consent preoperatively for the parent registry and additional written informed consent for the LIFELOG ancillary study.

### Additional Contributions

We thank all patients who participated in the SCRUM-MONSTAR LIFELOG study and the clinical staff who were involved in data collection at the National Cancer Center Hospital East.

### Previous Presentations

None.

## Supplement

Supplementary Material
