# Supplementary material for: Preoperative Patient-reported Physical Functioning and Postoperative Wearable-measured Physical Activity in Cancer Patients: A Secondary Analysis of the SCRUM-MONSTAR LIFELOG Study
Source: JMA J. 2026 Aug 14;9(5):1332–41. doi: 10.31662/jmaj.2026-0300 (PMC13639642; doi:10.31662/jmaj.2026-0300)

Supplementary Material

List of Abbreviations

**BMI**	Body mass index
**CI**	Confidence interval
**ENMO**	Euclidean Norm Minus One
**G8**	Geriatric-8 screening tool
**GGIR**	Generation of GeneRalized physical activity Indicators from Raw accelerometer data
**HR**	Hazard ratio
**IQR**	Interquartile range
**mg**	milligravity units (ENMO expressed in mg)
**MVPA**	Moderate-to-vigorous physical activity
**OW**	Overlap weighting (average treatment effect in the overlap population)
**PA**	Physical activity
**PF**	Physical Functioning
**pp**	Percentage points
**SF-36**	Medical Outcomes Study Short Form-36 health survey

Supplementary Table S1. Exploratory Univariable Cox Proportional Hazards Regression for Recurrence-Free Survival.

Variable	HR	95% CI	p Value
SF-36 PF (primary analysis)			
Impaired PF score (PF < 8) vs full PF score (PF = 8)	4.16	0.92–18.81	0.064
SF-36-derived PF composite score (per 1 point)	0.73	0.56–0.96	0.023*
Geriatric-8 (G8, comparison)			
G8-vulnerable (G8 ≤ 14) vs G8-fit (G8 > 14)	1.73	0.47–6.33	0.405
G8 total score (per 1 point)	0.87	0.67–1.13	0.290

p values from Wald test. *p < 0.05.
CI: confidence interval; HR: hazard ratio; PF: physical functioning; SF-36 PF: four-item subset from the Medical Outcomes Study Short Form-36 health survey Physical Functioning subscale.

Supplementary Table S2. Characteristics of Patients Grouped by Discordance between Preoperative Functional Assessment and Postoperative Physical Activity.

Characteristic	Under-performer (n = 40)	Concordant (n = 39)	Over-performer (n = 40)	p Value (under vs over)
Age, years	74.7 (67.0–79.8)	69.7 (58.5–76.9)	69.1 (59.9–76.5)	0.057
Male sex, n (%)	22 (55.0)	23 (59.0)	24 (60.0)	0.821
Cancer type, n (%)				0.835
Colorectal	8 (20.0)	10 (25.6)	10 (25.0)	
Urothelial	11 (27.5)	6 (15.4)	7 (17.5)	
Hepatocellular	4 (10.0)	7 (17.9)	2 (5.0)	
Renal cell	3 (7.5)	4 (10.3)	4 (10.0)	
Gastric	3 (7.5)	3 (7.7)	4 (10.0)	
Other	11 (27.5)	9 (23.1)	13 (32.5)	
SF-36-derived PF composite (0–8)	7.0 (6.0–8.0)	7.0 (6.5–8.0)	7.0 (6.8–8.0)	0.807
G8 score (0–17)	12.5 (11.5–14.1)	14.0 (13.0–15.0)	14.0 (12.5–15.0)	0.054
G8-vulnerable (G8 ≤ 14), n (%)	30 (75.0)	20 (51.3)	26 (65.0)	0.465
Disease stage (median)	3.0 (2.0–3.0)	2.0 (1.5–3.0)	2.5 (1.0–3.0)	0.467
Neoadjuvant chemotherapy, n (%)	6 (15.0)	6 (15.4)	8 (20.0)	0.770
Adjuvant chemotherapy, n (%)	22 (55.0)	20 (51.3)	23 (57.5)	1.000
Recurrence, n (%)	4 (10.0)	6 (15.4)	3 (7.5)	1.000
Observed mean acceleration (mg)	10.6 (7.5–13.4)	19.2 (16.8–22.0)	27.9 (25.5–40.0)	<0.001
Observed sedentary time (%)	92.4 (89.8–95.6)	85.1 (82.5–87.7)	78.4 (75.7–80.4)	<0.001
Observed MVPA (%)	1.5 (0.6–2.1)	3.5 (2.6–5.1)	6.8 (5.1–9.1)	<0.001

Groups are residual tertiles defined from linear regression of mean acceleration on the SF-36-derived PF composite score: under-performers (lowest residual tertile; observed activity below that predicted from preoperative function), concordant (middle tertile), and over-performers (highest tertile). Continuous variables: median (IQR); categorical variables: n (%). P values compare under-performers with over-performers (Wilcoxon rank-sum for continuous variables and Fisher's exact tests for categorical variables (sex, cancer type, chemotherapy, recurrence); disease stage was compared as an ordinal score (Wilcoxon rank-sum) and is summarized as the median. No adjustment was made for multiple comparisons. G8: Geriatric-8 screening tool; IQR: interquartile range; MVPA: moderate-to-vigorous physical activity; PF: physical functioning.

Supplementary Figure Legends

Supplementary Figure S1. Spearman and Kendall Correlations: G8 Total Score, G8 Domain Items, and Physical Activity Metrics. Each cell shows Spearman ρ (upper) and Kendall τ (lower). Top row: G8 total score (0–17), separated from the eight individual G8 domain items (below) by a thicker horizontal border. Bold borders around cells: $p < 0.05$ (Spearman). *$p < 0.05$; **$p < 0.01$; ***$p < 0.001$. N = 119. ρ: Spearman rank correlation coefficient; BMI: body mass index; mg: milligravity units; MVPA: moderate-to-vigorous physical activity.

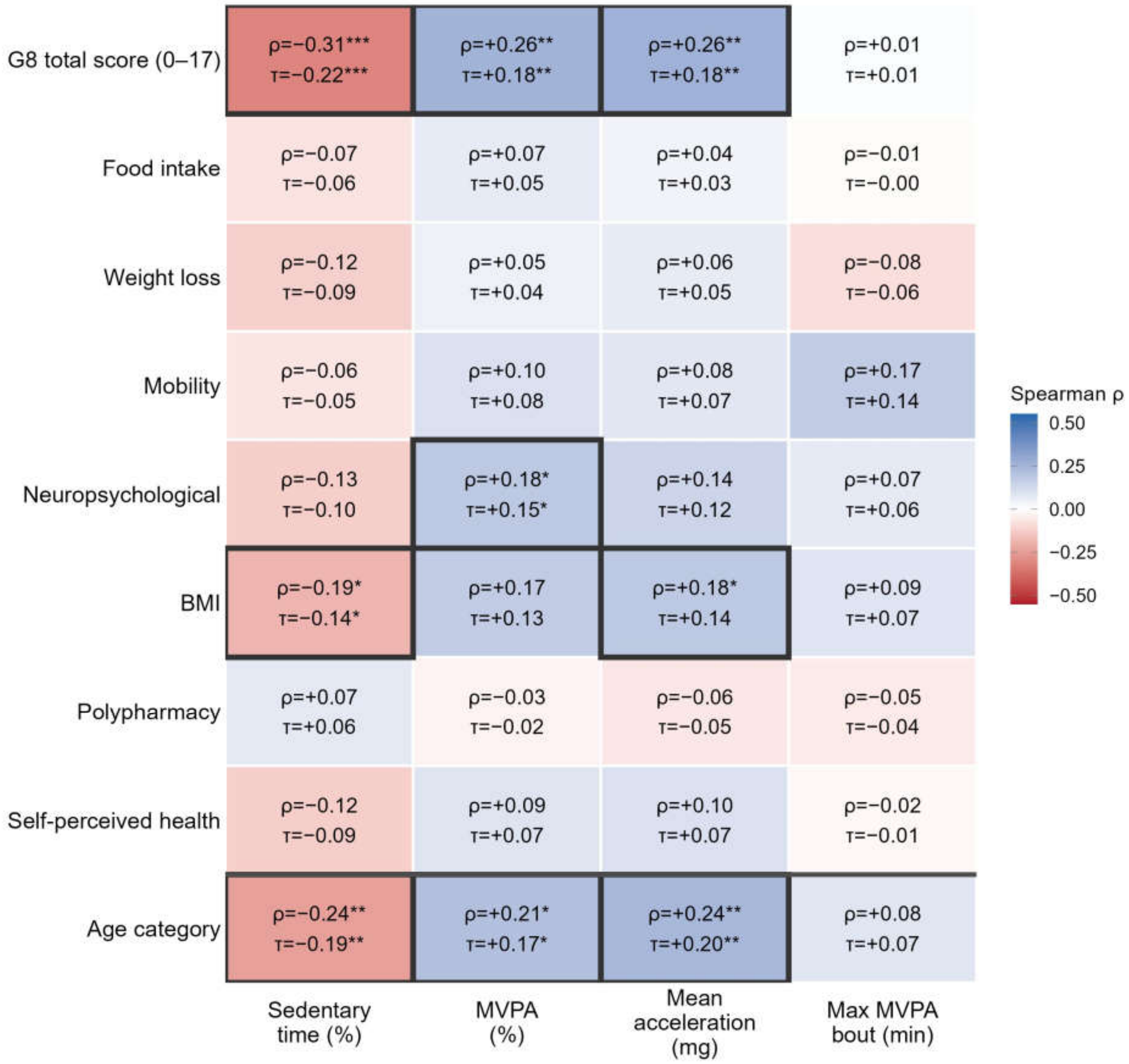

Supplementary Figure S2. Kaplan–Meier Recurrence-Free Survival Curves by G8 Category. Blue: G8-fit (G8 > 14); red: G8-vulnerable (G8 ≤ 14). Shaded areas: 95% CI. Log-rank p-value shown. Events = 13; median follow-up = 3.4 months (range 0.9–19.2). Exploratory analysis. CI: confidence interval; G8: Geriatric-8 screening tool.

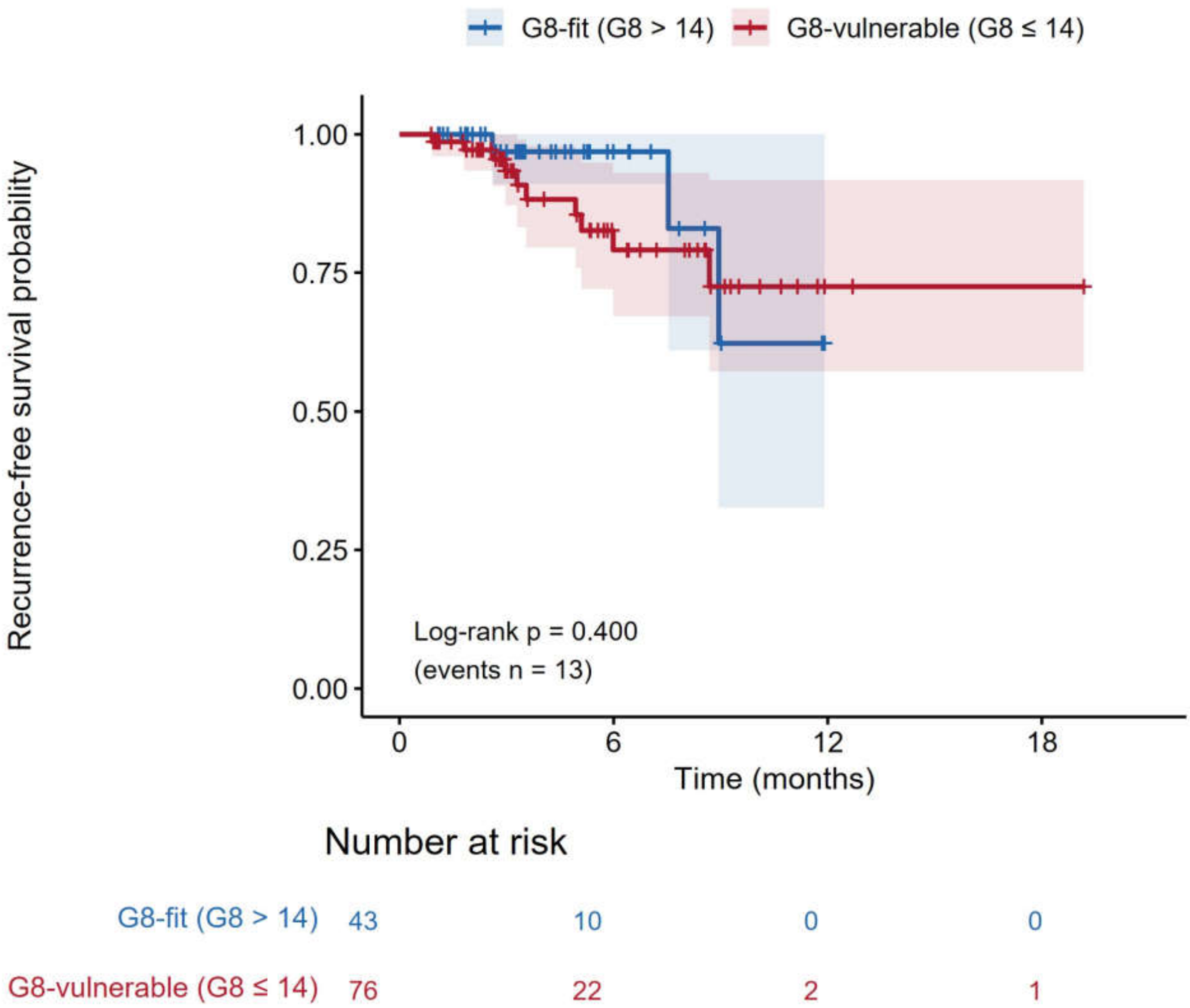

Supplementary Figure S3. Kaplan–Meier Recurrence-Free Survival Curves by PF Group. Blue: full PF score (PF = 8); red: impaired PF score (PF < 8). Shaded areas: 95% CI. Log-rank p-value shown. Events = 13; median follow-up = 3.4 months (range 0.9–19.2). Exploratory analysis. CI: confidence interval; PF: physical functioning.

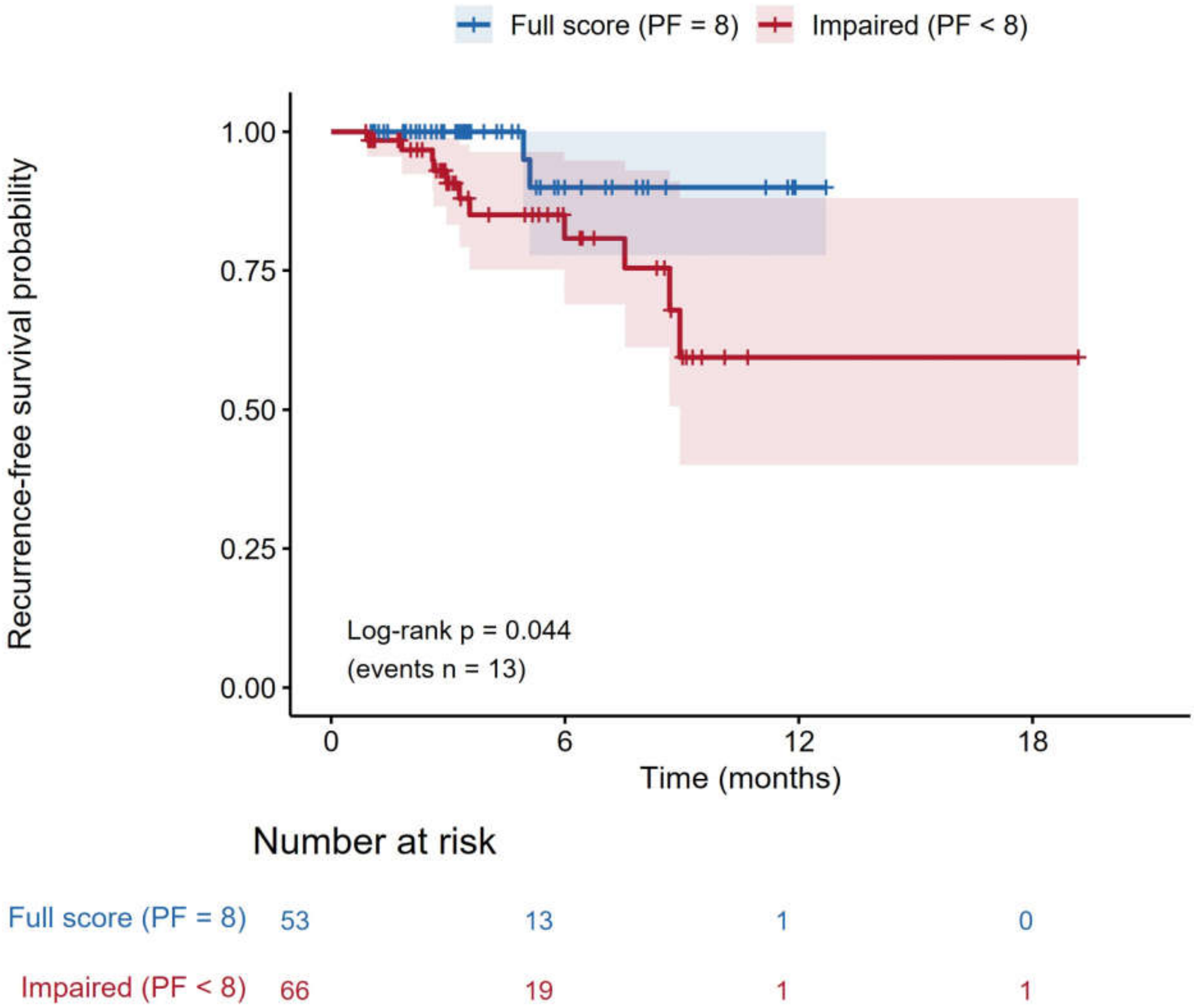

Supplementary Figure S4. Residual postoperative PA (observed minus predicted) versus age, for the three volume-related PA metrics. Residuals are derived from linear regression of each metric on the preoperative SF-36-derived PF composite score. For MVPA and mean acceleration, positive residuals indicate greater activity than predicted from preoperative function; for sedentary time, positive residuals indicate more sedentary time (i.e., lower activity) than predicted. The under-/over-performer tertiles used elsewhere are defined from the mean-acceleration residual. Each point is one patient (blue: full PF score, PF = 8; red: impaired PF score, PF < 8). Solid line: linear reference line with 95% CI (shaded); dotted line: zero residual. Spearman ρ and p (computed across all 119 patients) are shown per panel. Older age showed weak associations with lower-than-predicted activity across the three metrics. ρ: Spearman rank correlation coefficient; CI: confidence interval; mg: milligravity units; MVPA: moderate-to-vigorous physical activity; PA: physical activity; PF: physical functioning; SF-36: Medical Outcomes Study Short Form-36 health survey.

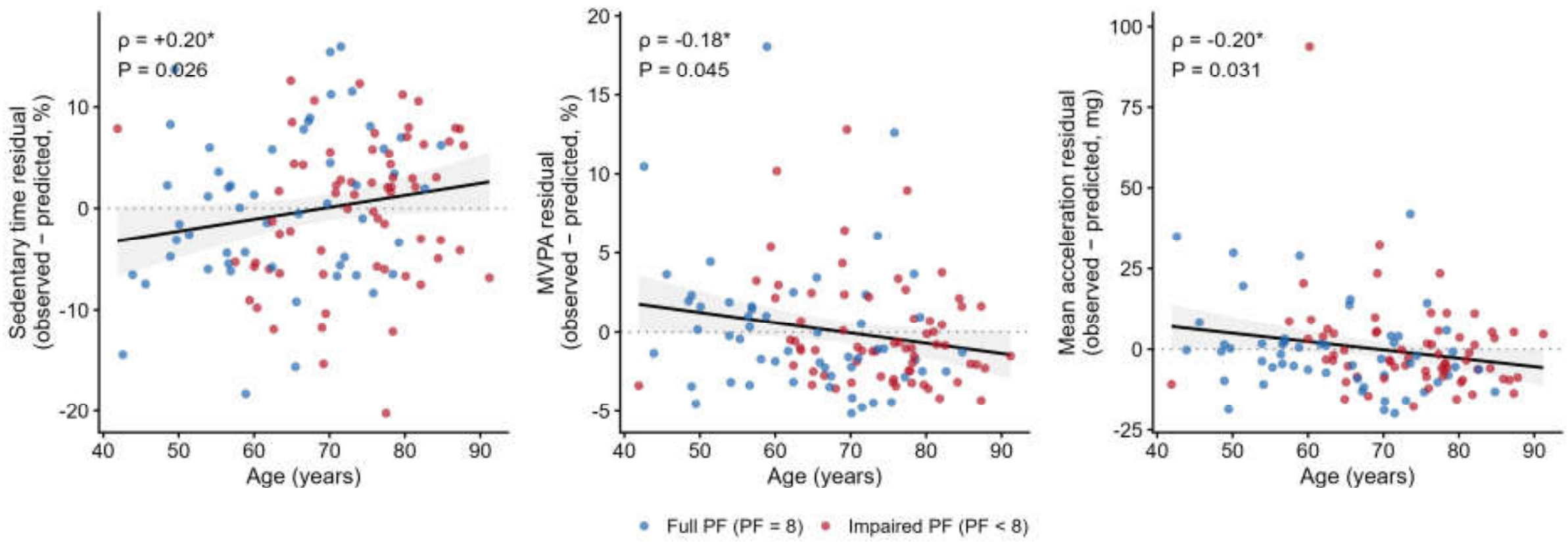

Supplementary Figure S5. Age and postoperative physical activity within the full–PF-score (ceiling) subgroup. Analysis restricted to the 53 patients reporting a full PF score (PF = 8), in whom the preoperative instrument cannot discriminate further because of the ceiling effect. Blue: full PF score patients (PF = 8; n = 53); grey: all patients (n = 119) shown for reference. Solid line: linear reference line with 95% CI (shaded), fitted within the full-PF subgroup. Spearman ρ and p are for the full-PF subgroup. A directionally similar but statistically non-significant age-related pattern was observed in this subgroup (mean acceleration ρ = −0.27, p = 0.054; sedentary time ρ = +0.22, p = 0.118); the estimates were imprecise and should be interpreted with caution. ρ: Spearman rank correlation coefficient; CI: confidence interval; mg: milligravity units; PF: physical functioning.

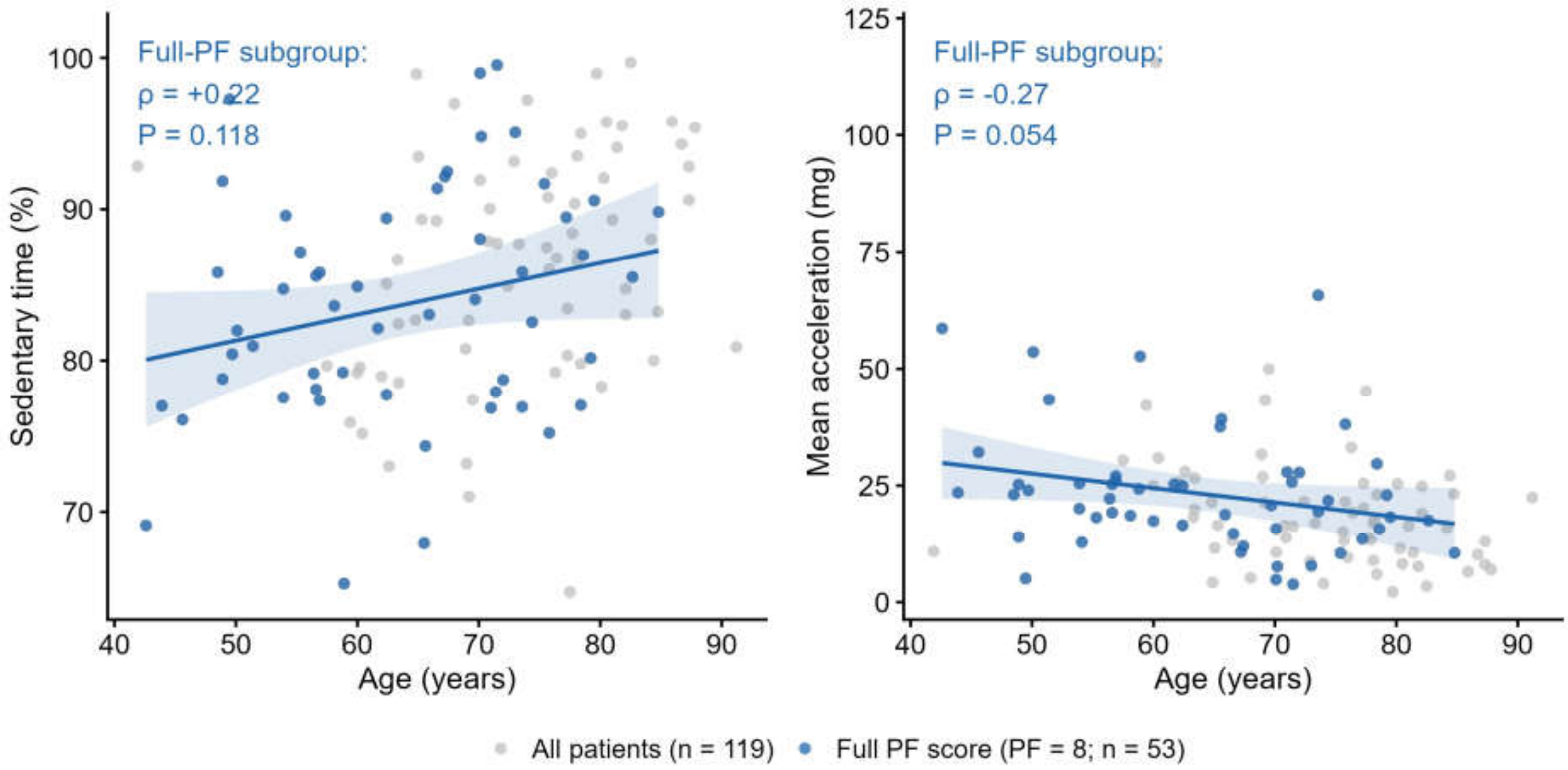

Supplement: Supplementary file 1 [file 2433-3298-9-5-1332-s001.pdf]
